# Dietary Treatment from Birth to Pregnancy in a Woman with Methylmalonic Aciduria

**DOI:** 10.3390/medicina57020128

**Published:** 2021-02-02

**Authors:** Agnieszka Kowalik, Anita MacDonald, Jolanta Sykut-Cegielska

**Affiliations:** 1 Department of Inborn Errors of Metabolism and Paediatrics, Institute of Mother and Child, 01-211 Warsaw, Poland; 2 Birmingham Children’s Hospital, Birmingham B4 6NH, UK; anita.macdonald@nhs.net

**Keywords:** methylmalonic aciduria, dietary treatment, childhood, pregnancy

## Abstract

Methylmalonic aciduria is treated with a natural protein-restricted diet with adequate energy intake to sustain metabolic balance. Natural protein is a source of methylmalonic acid precursors, and intake is individually modified according to the severity and clinical course of the disease. The experience and approach to MMA treatment in European centers is variable with different amounts of natural protein and precursor-free l-amino acids being prescribed, although the outcome appears independent of the use of precursor-free l-amino acids. Further long-term outcome data is necessary for early treated patients with MMA. This case study, a woman with MMA followed from birth to the age of 35 years, including pregnancy, illustrates the long-term course of the disease and lifetime changes in dietary treatment. A low natural protein diet (1.5 g–1.0 g/kg/day) was the foundation of treatment, but temporary supplementation with precursor-free l-amino acids, vitamin-mineral mixture, and energy supplements were necessary at different timepoints (in childhood, adolescence, adulthood and pregnancy). Childhood psychomotor development was slightly delayed but within the normal range in adulthood. There were few episodes of metabolic decompensation requiring IV glucose, but at age 27 years, she required intensive care following steroid treatment. In pregnancy, she remained stable but received intensive biochemical and medical follow-up. This successful long-term follow-up of a patient with MMA from childhood, throughout pregnancy, delivery, and postpartum confirms that careful clinical, biochemical, and dietetic monitoring is crucial to ensure a favourable outcomes in MMA. Personalized treatment is necessary according to the individual clinical course. Knowledge about long-term treatment and clinical outcome is important information to influence future MMA clinical guidelines.

## 1. Introduction

Methylmalonic aciduria (MMA) is an autosomal recessive rare inherited disorder of l-isoleucine (ile), l-methionine (met), l-threonine (tre) and l-valine (val) metabolism caused by the defect in pathway of conversion of methyl-malonyl-CoA to succinyl-CoA. The severe presentation of intoxication syndrome may occur early in the first days of life or late in childhood or adolescence [1]. In both situations the treatment should commence without delay, even if the diagnosis is not yet confirmed. The principle of dietary treatment is reduction or elimination of toxic metabolites by lowering daily protein intake and providing adequate energy intake. Medical management includes carnitine supplementation (50–100 mg/kg/day) to prevent secondary carnitine deficiency, and antibiotics (metronidazole) to reduce propiogenic gut flora. In the case of hyperammonemia, treatment with sodium benzoate (which is controversial) or preferably with carglumic acid may be required [2]. Dietary treatment and pharmacological treatment are necessary throughout life. Restriction of natural protein, a source of methylmalonic acid precursors, is individually modified, depending on the severity and clinical course of the disease. The experience and approach to MMA treatment in European centers is variable with different amounts of natural protein with or without precursor-free l-amino acids being prescribed [3,4]. Overall, outcome appears independent of the use of precursor free l-amino acids and recent reports even suggest adverse effects associated with high leucine containing precursor free l-amino acid formula in MMA [5]. Although there are European guidelines for the dietary management of MMA [2], further long-term outcome data is necessary. Our case study is the first diagnosed case of MMA in Poland, which illustrates the long-term course of the disease and changes in the dietary treatment approach required throughout life. 

## 2. Case Report

### 2.1. Development and Clinical Status

A female patient, now 35 years old, was the second child but the third pregnancy from consanguineous parents. She was born at term, with a birth weight of 2890 g, length of 58 cm, and head circumference of 33 cm. The Apgar score was 10 points. During the first 4 months, vomiting, diarrhea, and metabolic acidosis occurred. At age 6 months, isolated methylmalonic aciduria was diagnosed based on the organic acid profile analyzed by the gas chromatography combined with mass spectrometry method. Treatment with cobalamin appeared ineffective, and MMA B12 unresponsive form was established (genetic testing was unavailable at the time of diagnosis). 

Since then, the patient has been treated and monitored in a conventional way. Our patient was maintained on a protein-restricted diet (approx. 1.5 g/kg/day), mainly of low biological value, but some high biological foods were recommended. During childhood, there were recurring episodes of metabolic decompensation, usually associated with infections and increased energy expenditure. Therefore, she required hospitalization (on average 2–3 times per year in the first years of life), treated with intravenous glucose infusions and an increased dosage of l-carnitine. Measurements of the urinary methylmalonic acid were 533 μmol/mmol creatinine at age 2 years, 8930 μmol/mmol creatinine at age 6 years, and 27,206 μmol/mmol creatinine at age 7 years. Poor appetite and food refusal persisted. Her psychomotor development was slightly delayed. She could sit unsupported at age 12 months and walked at 16 months. After diagnosis and introduction of treatment with protein restriction, her somatic development significantly improved up until the age of 3 years. In the following seven years, deceleration in her growth and body weight was evident, as illustrated Table 1. At 11 years, z-score for weight was −1.19 and height was −1.84. During puberty, menses commenced at 15 years and z-score for weight and height reached −1.53 and −1.19, respectively. At age 18 years, BMI was 19.2 and z-score for weight and height improved at −0.81 and −0.65, respectively. At the same age, hepatitis type C was diagnosed and treated with interferon at age 23 years. Unexplained leg pain and weight loss of 6 kg were observed and during this period she had a three-fold increase in MMA in the urine. However, generally during her childhood and adolescence no known MMA complications, such as kidney dysfunction, pancreatitis, or cardiomyopathy, were identified. The most life-threatening episode occurred at the age of 27 years, when steroids were prescribed for ophthalmologic complications. She was admitted to intensive care unit and required dialysis. In the following four years, she remained stable but without strict metabolic control as she was lost to follow-up.

### 2.2. Dietary Treatment

In her first 10 years, she had a restricted natural protein intake, 1.4–1.5 g/kg body weight (b.w.) with animal protein providing 40% of intake. In the second decade of life, deceleration of somatic development was observed, which was related to inadequate energy and natural protein intake, so precursor free amino acids (threonine, methionine, valine, and isoleucine) were added. The diet supplied 1 g/kg b.w. protein equivalent and natural protein intake was maintained at 0.85 to 1.6 g/kg b.w. between 11 to 14 years. From the age of 15 to 19 years, the protein equivalent of l-amino acids decreased to 0.4 g/kg b.w. and the intake of natural protein was maintained in the range of 0.9–1.0 g/kg b.w. From 20 to 30 years, natural protein intake ranged from 0.6–1 g/kg b.w, and protein equivalents of precursor-free l-amino acids, 0.4–0.3 g/kg b.w and energy intake were maintained at 1850 kcal/day. Additional vitamin B12 was supplemented intramuscularly (at the insistence of the patient’s mother): once weekly in the first year, once every two weeks, aged 1 to 5 years, once monthly aged 6 to 10 years, and every 5–6 weeks up to 18 years. The supplementation was stopped at age 18 years. 

### 2.3. Pregnancy 

At the age of 31, she became pregnant, and monthly clinic follow-up visits and additional biochemical tests were established. Every month, the amino acid assay, ammonia, organic acid profile in the urine, and acylcarnitine profile in dried blood spots (DBS) were monitored. MMA and methyl-citrate were measured in DBS regularly.

In the first trimester of pregnancy (first visit), the daily energy intake was increased to 2300 kcal, and natural protein to 1.0 g/kg b.w. Hyperglycemia of pregnancy (glucose > 98 mg/dL) was observed, which resolved after the lowering of simple sugars’ intake. However, during the first six months of pregnancy, vomiting was common, and in the seventh month of pregnancy she required 10% intravenous 10% glucose (for 8 h). The patient had a proximal humerus fracture at 6 months of pregnancy, which restored without any complications. For four weeks, natural sources of collagen were included as part of her controlled protein intake together with frequent regular plasma amino acid concentrations with the aim of maintaining an amino acid profile within the reference range. Her appetite was poor with energy and protein intake inadequate. A high protein supplement (Protifar^®^, Nutricia company, Zoetermeer, The Netherlands) and high-protein yogurt were included as part of her natural protein allowance. To increase the energy content, a glucose polymer (Fantomalt^®^, Nutricia company) and a protein-free formula (Milupa basic-p^®^, Nutricia company) were added. Intake of energy, natural protein, and total protein increased in the following months, sustaining a constant supply of natural protein intake throughout pregnancy. Due to the persistent low plasma concentration of isoleucine (<20 μmol/L) and valine (<50 μmol/L), supplementation with these single amino acids was included in a dose of 100 mg per day in the first trimester and 150 mg of each in the second and third trimester of pregnancy. Fetal ultrasound, which was performed three times, was normal. Her weight gain was 12.5 kg (from 1 kg to 5.5 kg and 6 kg in the first, second, and the third trimester, respectively). The dose of carnitine was modified, starting at 3 g/day in the first trimester and increasing to 4 g/day in the second and third trimester. There were no clinical or biochemical signs of metabolic decompensation during pregnancy.

### 2.4. Delivery and Postpartum Period

A healthy girl was born at 38 weeks of pregnancy by C-section (weight 3280 g, length 52 cm, Apgar 10 points). In the perinatal period there was no metabolic decompensation, although there was an elevation of MMA to 31.1 µmol/L in DBS two weeks before delivery. To avoid catabolism, the patient received continuous glucose by 10% intravenous infusion and intravenous carnitine one day before, during, and two days after delivery, and from the second day precursor-free l-amino acids were given. From the fifth day post-delivery, the patient consumed her full energy and protein prescription (2200 kcal/day, protein 1.2 g/kg, respectively) with isoleucine and valine supplementation. She breastfed and a high-energy diet was recommended. After four weeks, her daily energy intake was 2600 kcal and protein intake 1.68 g/kg b.w, respectively, with 300 mg/day of either isoleucine or valine supplementation. The baby has been developing well without any clinical problems.

### 2.5. Current Diet

The patient with MMA breast fed for 6 months and during this time her body weight decreased by 5 kg. Energy and protein intake were low. A natural protein intake of 0.8–1.0 g/kg b.w. was recommended. The l-amino acid supplement was replaced with vitamin and mineral supplementation. The isoleucine and valine supplementation was reduced to achieve metabolic plasma amino acid balance and her final plasma levels of isoleucine were 27 µmol/L and valine, 81 µmol/L (reference range for age >16 years; isoleucine 34–84 µmol/L, valine 144–269 µmol/L). Efforts to care for the baby and persistent poor appetite led to a weight loss of 5 kg. She did not eat regularly, but used high-energy drinks when she was tired. 

## 3. Discussion

The dietary treatment of MMA restricts natural protein. Although the first experiences and therapies regarding dietary MMA treatment were published four decades ago, many questions remain [6,7,8,9]. Natural protein tolerance varies, and the severity of exclusion of methionine, threonine, valine, and isoleucine depends on the form and severity of the disease [3]. Since early childhood, our case had a poor appetite. In her early years of life, the intake of natural protein (around 1.5 g/kg b.w.) met safe levels of protein intake for the development and maintenance of metabolic balance (FAO/WHO/UNU 2007) [10]. A formula-free of propiogenic amino acids was initially unnecessary. Observations by Touati et al. indicated that infants fed without a special formula had a better plasma amino acid profile [3]. However, in the following years, our case had a development delay, which was possibly related to inadequate energy and nutrient intake, and interruption of nutrient intake with illness management. A clear improvement in growth and physical development occurred after the introduction of precursor-free l-amino acids, which also increased the daily supply of vitamins and minerals, including calcium and iron that met the daily requirements (RDA) recommended for age. In MMA, the risk of osteopenia and osteoporosis is high. North et al. showed improvement in bone following the introduction of gastrostomy feeding in a patient with MMA and osteoporosis [11]. In our patient, at the age of 20 years, osteopenia was diagnosed, which was treated with Ca and vitamin D supplementation with good effect. In adulthood (27–30 years), she was lost to follow-up, so biochemical and dietary control was irregular. At the age of 27 years, there was severe, life-threatening metabolic decompensation triggered by steroid treatment. At the beginning of pregnancy, her BMI was 19.1 and the intake of both energy and protein were borderline safe and required immediate increase, to minimize the risk of decompensation and fetal malnutrition. Pregnancy is an anabolic period generates a higher need for nutrients [12]. Glucose drinks were used to meet energy requirements but due to hyper-glycaemia had to be replaced by other energy sources, especially in the first trimester of pregnancy. Persistent anemia up to the third pregnancy trimester could have impacted on appetite reduction and food aversion. In the later months of pregnancy, the increasing need for protein was supplied by dairy products and Protifar^®^ as well as a small amount of sausage and chicken pates. Intake of energy and protein throughout pregnancy was satisfactory, without any risk to fetal development. The intake of natural protein was increased in line with the weight gain of the pregnant woman in the subsequent stages of pregnancy, from 40 g/day in the first trimester to 50 g/day in the second and a maximum of 80 g/daily in the last trimester. Similar amounts were prescribed in other reports [13]. The case of a newborn with intrauterine dystrophy was reported by Langendonk et al. as a complication of energy protein deficiency in a pregnant woman with MMA [14]. In our case, there was no need to use additional precursor-free l-amino acids during pregnancy, but l-isoleucine and l-valine were supplemented. We monitored biochemical parameters and fetal ultrasound to ensure optimal control and safety for both the mother and the baby. The mother was metabolically stable post pregnancy. She received carnitine throughout pregnancy, similarly to 7 out of 13 other pregnant women with MMA [13]. A high dose of carnitine (4 g/day) was also maintained during breastfeeding for six months. In the postpartum period, there was a slow return to tolerated dietary protein intake, considering the risk of catabolic conditions associated with uterus involution. At this time, the preventive glucose intravenous infusion was included, as with other described cases [12,13,15]. The postpartum period was uneventful with a hospitalization time of ten days [16,17]. Her daughter at the age of three years is healthy, with normal psychomotor development [13,18]. Management required close cooperation between the physician, dietitian, and the patient, with frequent dietary analysis and careful monitoring of energy intake, specially. During pregnancy, dietary management requires frequent systematic modification, considering patient well-being, food tolerance, and taste preferences. 

## 4. Conclusions

This successful long-term follow-up of this patient with MMA from childhood until pregnancy, delivery, and the postpartum period confirms that careful clinical, biochemical, and dietetic monitoring is crucial to ensure a favourable outcome in MMA. Treatment should be personalized according to the individual clinical course. Increasing knowledge and experience about long-term outcome data of patients at different life stages is required for the development of therapeutic guidelines in MMA. 

## Figures and Tables

**Table 1:** Z-scores of the patient’s body weight and height, and content of total, natural and equivalent protein in the diet.

Age	Z-Score of Body Weight	Z-Score of Body Height	Total Protein (g/kg)	% of Natural Protein	% of Equivalent Protein
3 months	−3.33	n.a	n.a (before diagnosis)		
4 months	−2.25	n.a	n.a (before diagnosis)		
9 months	−1.89	−2.75	1.5	100	0
12 months	0.50	−1.27	1.5	100	0
16 months	1.93	−0.36	1.5	100	0
22 months	1.67	n.a	1.5	100	0
24 months	1.5	0.06	1.5	100	0
30 months	1.0	−0.83	1.4	100	0
3 years	0.47	−0.88	1.4	100	0
4 years	−0.21	−1.14	1.4	100	0
5 years	−0.2	−1.06	1.4	100	0
7 years	−0.75	−1.21	1.4	100	0
8.5 years	−0.94	−1.24	1.4	100	0
11 years	−1.19	−1.84	2.6	60	40
13 years	−0.89	−1.89	1.9	50	50
14.5 years	−1.63	−1.48	2.5	60	40
15 years	−1.53	−1.19	1.3	70	30
16 years	−1.15	−1.04	1.4	70	30
17 years	−0.85	−0.77	1.4	70	30
18 years	−0.81	−0.65	1.3	70	30

n.a.—data unavailable.

## Data Availability

The data reported in this paper are available from the medical history of the patient.
